# Assessment of the TMJ Dysfunction Using the Computerized Facebow Analysis of Selected Parameters

**DOI:** 10.1155/2015/508069

**Published:** 2015-05-11

**Authors:** Edward Kijak, Danuta Lietz-Kijak, Bogumiła Frączak, Zbigniew Śliwiński, Jerzy Margielewicz

**Affiliations:** ^1^Department of Prosthetic Dentistry, Faculty of Medicine and Dentistry, Pomeranian Medical University, Rybacka 1, 70-204 Szczecin, Poland; ^2^Department of Dental Propedeutics and Physiodiagnostics, Pomeranian Medical University, Rybacka 1, 70-204 Szczecin, Poland; ^3^Institute of Physiotherapy, Jan Kochanowski University, Żeromskiego 5, 25-369 Kielce, Poland; ^4^Silesian University of Technology, Krasińskiego 8, 40-019 Katowice, Poland

## Abstract

*The Purpose of the Paper.* Qualitative and quantitative analysis of selected parameters of mandible movements, electronically registered in patients with temporomandibular joint dysfunction and healthy ones.* Material*. Function test of the mandible movements was conducted in 175 patients. Gender distribution was 143 women and 32 men, aged 9 to 84. * Methods*. The studied population, after accurate clinical examination, was divided into age groups with the range of five years. All the patients had Zebris JMA computerized facebow examination done, according to the generally accepted principles and procedures.* Results.* Mean values of mouth opening calculated to 45.6 mm in healthy group and 37.6 mm in TMJ dysfunction group. Mean length of condylar path amounted to 39 ± 7% of the maximum value of mouth opening in the group of healthy people, 44 ± 11% in the case of muscle-based disorders, and 35 ± 11% with joint-based. The mean value of the condylar path inclination oscillated in the range of 25° to 45°.* Conclusions*. The ratio of length of the condylar path to the size of mouth opening may be a significant value characterising the type and degree of intensification of the TMJ dysfunctions.

## 1. Introduction

Joint-muscle and dental system dysfunctions, often also known as the locomotor system function disorders (LSFD), are a serious diagnostic and therapeutic difficulty in dental practice [1–5]. In the recent years the number of people with pain in the area of head and neck increased [6]. According to the latest tests conducted in highly-developed countries, it is assumed that even approximately 75–90% of the population suffer from temporomandibular disorders, according to Carlsson [7], Macfarlane et al. [8], and Rugh and Solberg [9]. The basic symptoms of the TMJ dysfunctions are pain during mandible movements, limitation of its mobility and related hindered or painful mastication, clicking in temporomandibular joints during movement, masticatory organ muscles hypertension, headaches, and cervical pain. Frequently, dysfunctions are accompanied by various types of parafunctions, for example, bruxism [10–13]. The factors generating the temporomandibular disorders include mental stress [14], bad habits, acute and chronic injuries, incorrect muscles functioning, traumatic occlusion, iatrogenic factors, mental disorders, and hormonal disorders, as well as generalised joints diseases, which was confirmed by Greek [15], LeResche at al. [16] and Egermark-Eriksson et al. [17] and Curcic [18]. Temporomandibular joints are most often used joints in the human body. They also participate in many physiological activities, such as speech, receiving food, singing, yawning, and even expressing emotions. It has been observed that within a day dental arches are in contact for ca. 30 min—mainly during swallowing saliva. Incorrect teeth contact caused by, for example, tooth loss, bruxism, and nontreated dental caries are the source of stresses in temporomandibular joints, which sometimes can lead to activation of a cascade of unfavourable events, which as a consequence leads to a serious disorders. The purpose of the paper was qualitative and quantitative analysis of selected parameters during mandible movements, registered in patients with TMJ dysfunctions and healthy ones. Clinical tests were conducted by computerized Zebris JMA facebow.

## 2. Material 

**Figure 1:**
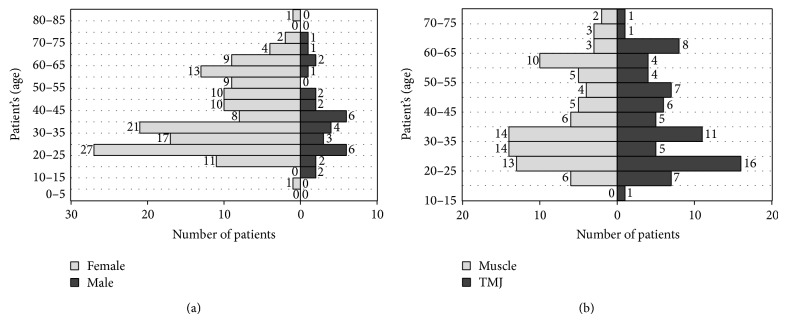
Characteristics of the studied population owing to age: (a) the number of people in groups of examined patients with the distinction of gender, (b) cause dysfunction of the mastication organ.

Function test of the human mastication organ was conducted in 175 patients: 143 women and 32 men aged 9 to 84, who, in the period of the last 7 years, volunteered to the Department of Prosthetic Dentistry of the Pomeranian Medical University in Szczecin because of disorders in the mastication organ functions. A reference material was a group of 13 potentially healthy people, that is, ones who reported no ailments, and the results of conducted clinical tests have not demonstrated symptoms of a human mastication system dysfunctions. Graphic records, reflecting the stomatognathic system functioning in healthy people, were recognised as a model. From anamnesis in every patient the following was recorded: age, height, body weight, and opinions expressed with regard to symptoms and ailments. The studied population was divided into age groups with the range of five years. The average age of the tested group of women was 38.6 and standard deviation was equal to 15.95. With regard to the group of men, the average age was 36.25 and standard deviation was equal to 15.68. The oldest man was 73 years old, while the youngest was 12. Then, additional three groups were separated. The first one was represented by 76 patients: 60 women and 16 men in whom the clinical test diagnosed improper operation of the temporomandibular joints. The second group qualified 86 patients, including 75 women and 11 men, with diagnosed improper mastication organ muscles functioning. The last third group of 13 tested were healthy people, that is, not reporting any ailments. The average age of the people was close for each of the separated groups and was accordingly: for the sick with joint basis of disorders ca. 37.9, with muscle troubles 39.5 and 31.3 in the case of healthy people. Differences in numbers of particular age groups and the participation of women and men in the surveyed population are presented in Figure 1(a).

On the basis of the conducted analyses, it was determined that the mastication organ functioning disorders occur over 4 times more often in women than men. The diagram shows that from the point of view of source of the disorder: joint or muscle dysfunctions is a feature not statistically important, because the conditions coming directly from joints occur equally often as the muscle cause of disorders and are independent from age of the studied persons (Figure 1(b)).

## 3. Methodology of Research

The clinical test was conducted according to the generally binding principles and any standard procedures for this type of cases. The symptoms of function disorders were assessed as well as the level of condition progress: presence of spontaneous pain disorders in the surroundings of the stomatognathic system of the facial part of the cranium, their location, and duration. The clinical diagnostics was mainly aimed at determination of the nature and grounds of disorders: joint TMD (Temporomandibular Disc Displacements) or muscle (Muscle Disorders). Assignment to groups was based on the generally accepted diagnostic protocol RDC/TMD. The first group—the joint—are persons who meet the criteria of groups IIa and IIb of the said classification. The second established a person with symptoms of belonging to both groups—Ia and Ib muscle diseases by the RDC/TMD.

**Figure 2:**
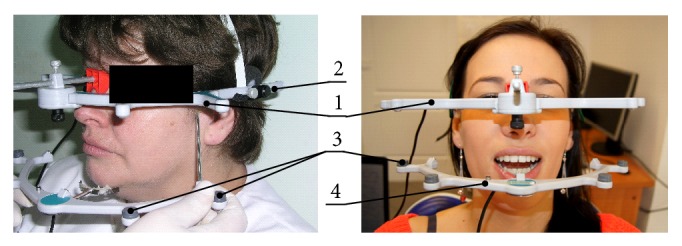
Electronic Zebris JMA facebow, graphic imaging of procedure of introducing measuring points, and assembled device ready for use.

The clinical test in most cases is insufficient and must be supplemented with additional tests with the use of specialist apparatus. Such action is necessary, because the classic diagnostics, conducted usually with the data from anamnesis and physical examination, visual, auscultation and palpation, is often insufficient. Therefore, in order to precisely formulate the diagnosis, in the least burdensome manner for the patient as well as the doctor, patients underwent electronic assessment of efficiency of the mastication organ with the Zebris JMA device (Figure 2).

Zebris JMA is a complex recording system, computer-controlled, whose spectrum of applications in functional diagnostics has been significantly extended. The apparatus has two stiff measurement arches, whose installation in a proper position is definitely simplified. The face ring, upper arch (1), is put on the nose and fastened at the back of the head over the ears using a plastic belt (2). Measurement sensors (3) are located in the mobile arch (4) which must be precisely fixed to the labial surface, front teeth of the mandible. Additionally, connection of the sensor with the teeth cannot disturb when recording functional movements of proper intercuspidation. Low weight of the lower arch amounting only to 20 g does not tire or overload the patient. The sensors located in the lower, mobile arch record change in intensity of ultrasonic waves, generated with the frequency of 900 Hz, through immobile transmitters located in the upper arch. The results are precise, recorded with the accuracy of ±0.1 mm, three-dimensional trajectories, on which the heads of condyloid process and incisors move. The course of the examination is relatively short and simple. After the installation of measurement arch, the patient performs a dozen or so mandibular movements. At this point, the sensory system records trajectories: abduction and adduction, double-sided laterotrusive movements and protrusive movements; the computer saves the real-time measurement data. An important function of the discussed apparatus is the possibility to introduce additional orientation points, characterising the individually variable characteristics of geometric structure of the facial part of the patient's cranium, the effect of which is a multiparameter analysis of occlusion and joints operation. According to the authors of such registration, it can be done using any electronic device of similar effect, which has the ability to individualize research—introducing arbitrary points. At this point, the following should be listed: for example, optoelectronic computer systems (Condylocomp, Cadiax), devices using for recording mandibular function movements ultrasonic sensors (Zebris JMA, Arcus digma), and magnetic (K-7). These are complex recording systems, computer-controlled, where the spectrum of applications in functional diagnostics has been significantly extended. Discussed system was used only because of its availability.

## 4. Test Results: Results of Statistical Analysis

During statistical analyses performance, the following statistical tests were used: Kolmogorov, Kolmogorov-Smirnov, and Test for Homogeneity of Variances, which was preceded by the Bartlett test. All necessary calculations were made assuming the levels of significance: *α* = 0.01 and *α* = 0.05. Statistical analyses started from verification of the tested population, in terms of application of homogenous objective and subjective data assessment criteria, collected in clinical conditions. Special attention was paid to checking whether the populations of women and men form normal distributions and are homogeneous in terms of age. The conducted Kolmogorov statistical tests proved, at the assumed level of significance *α* = 0.01, that the tested groups of women and men are normal distributions. We can believe so, since the calculated statistics values *λ* amount to, respectively, 1.548 and 0.701 and are smaller than the critical value *λ*_0.01_ = 1.627. The Kolmogorov-Smirnov equality test clearly demonstrated *λ* = 0.64 that both groups are homogeneous in terms of age of the tested persons, and thus they can be analysed together.

Still on the basis of graphic records of the recorded measurement data, detailed assessment covered the following parameters: the scope of mouth opening gaps *Y*, namely, the degree of mandible abduction, that is, maximum dimension measured between the incisal edges of central incisors; the length of articular route—the scope of movement of condyloid process heads in abduction movements *S*_*i*_ and a measurable parameter being inclination angle of the articular route* SCI*.

**Figure 3:**
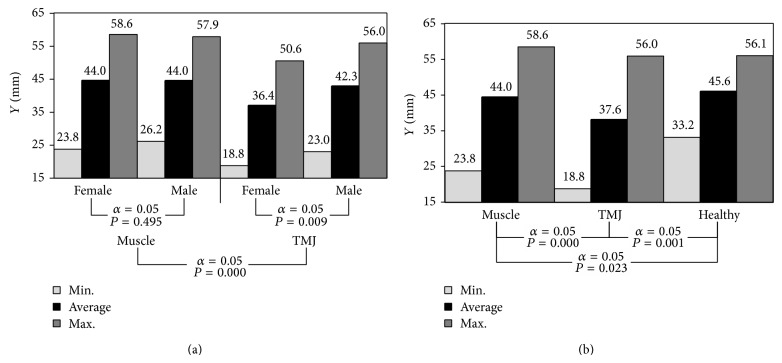
The diagrams illustrating the maximum opening gap depending on (a) gender and (b) source of dysfunction.

The graphically pictured data show that there are no statistically significant differences between the maximum values of mandible abduction in the group of women and men (Figure 3(a)). Average values of the analysed parameter were accordingly, for healthy people 45.6 mm, for people with joints functioning dysfunction 37.6 mm and muscles 44 mm (Figure 3(b)).

**Figure 4:**
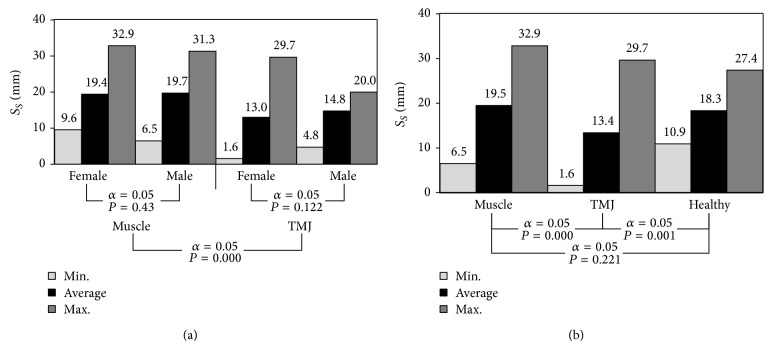
The diagrams illustrating the route of condyloid process heads depending on (a) gender and (b) source of dysfunction.

**Figure 5:**
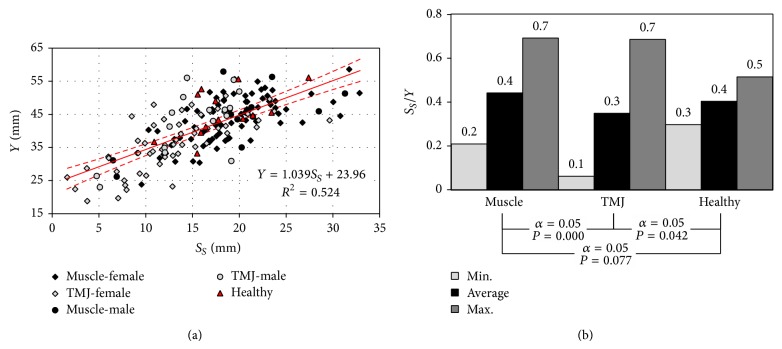
(a) The relation occurring between maximum dental arches opening *Y* and the average articular route *S*_*S*_ and (b) diagram showing statistics calculated on the basis of the relation and average articular route to the maximum dental arches opening.

The diagram (Figure 4(a)), presents the statistical values of the articular route in particular examined groups. Similarly as in the analysis of maximum dental arches opening *Y*, so in the case of the articular route *S*_*S*_, reference of the analysed parameters to healthy patients indicates that, in people complaining about ailments, whose source is incorrect joints and muscles operation, a substantial reduction in the length of the articular route is observed (Figure 4(b)). The highest values of the length of the articular route were recorded in the group of women with muscle etiology of the disorder. This issue is better illustrated by a collective graph of dependencies between the average length of the articular route *S*_*S*_ and the size of opening gap *Y* (Figure 5(a)).

The diagram (Figure 5(b)) statistics describing the relation of average length of the articular route *S*_*S*_ to maximum dental arches opening gap *Y* were compared.

**Figure 6:**
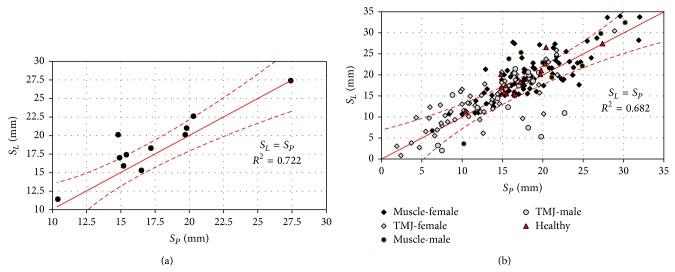
The causal relation existing between the average length of the right and the left articular route and in the abduction movement: (a) in healthy patients and (b) all the examined patients.

The operation of both joints is well illustrated in the graph of relation between the length of the right *S*_*P*_ and the left *S*_*L*_ articular route in healthy individuals (Figure 6(a)). The distribution of data included in Figure 6(b) indicates that the relation between the average lengths of articular routes, the right and left condyloid process in healthy patients and with disorders, oscillates within the approximating straight line.

**Figure 7:**
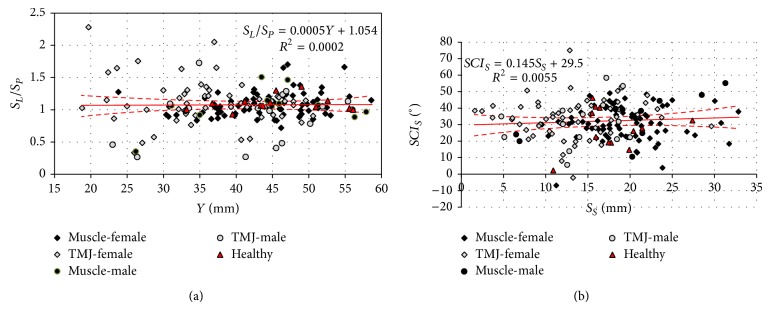
Relation characterising the relation between (a) the relation of the length of articular routes in joints and the maximum opening gap and (b) average articular angle* SCI*_*S*_ and the average articular route *S*_*S*_.

The data included in the chart (Figure 7(a)) show great compliance of the concerned parameter in the majority of examined people as compared to the healthy people. However, the largest deviations are observed in the group of men and women with joint disorders. Among measurable kinematic sizes, one of the most essential is the so-called inclination angle of the articular route* SCI*, which was referred to the average articular route (Figure 7(b)). Its value, on average, is a few dozen degrees and is in fact the route that the condyloid process head covers during abduction and adduction of the mandible.

**Figure 8:**
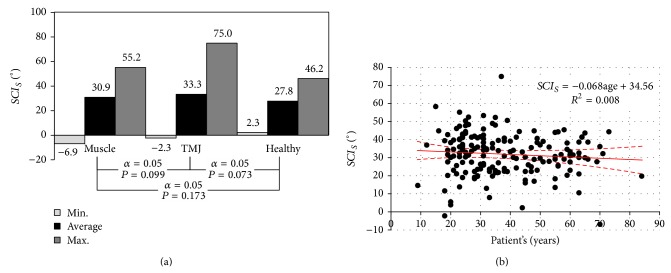
The relation characterising the relation of the average articular angle: (a) diagram showing the statistics in particular groups of studied population and (b) the age of the patient.

The conducted statistical analyses indicated that the lowest average value of articular angle in the examined population occurs in the group of the sick having dysfunction of articular etiology (Figure 8(a)). The obtained results prove unambiguously that the average value of the inclination angle of the articular route* SCI*_*S*_, oscillates between 25 and 45° (Figure 8(b)).

## 5. Discussion

An important role in LSFD diagnostics apart from anamnesis and physical examination is played by the image examination: Panoramic X-rays, as check-up examination, computer tomography techniques (CT), and magnetic resonance (MR) and axiographic evaluation. Direct imaging methods have already been discussed many times for example, by Tanimoto et al. [19], Görgü et al. [20], Wong et al. [21], Sukovic [22], and many others. In recent years many researchers emphasise the importance of electronic axiography in differential diagnostics of mastication organ dysfunctions, due to accuracy and precision of the measurement data obtained, Celar and Tamaki [23] and Pröschel et al. [24]. The use in daily clinical practice of modern, often complex diagnostic techniques is becoming necessary, especially with regard to patients with intensified symptoms of stomatognathic system functions disorders, often accompanied by morphological changes [8, 9, 24, 25].

Research results obtained by various researchers indicate the main difference in the scope of dental arches opening in the group of women and men. Men on average by ca. 5 mm open their mouths broader as compared to women, as indicated in the paper [26]. On the other hand, the results of latest research conducted with the group of 12 men and 15 women, aged 19 to 30 determine this difference at the level of approximately 10 mm [27]. It is believed that a typical scope of maximum dental arches opening in the group of men is within the range from 50 to 60 mm and women from 45 to 55 mm [28]. The size of an opening gap is one of the parameters indicating the degree of intensification of mastication organ dysfunction, which is determined, for example, on the basis of the Helkimo index [29]. Literature reports indicate that the range of mouth opening changes with age of the body. Additionally, in the initial period of life it grows [30] until achieving maturity. At the time of achieving maturity the possibility of broad mouth opening is becoming gradually limited [31].

On the basis of the conducted statistical analyses of population with muscle etiology, it has been stated that the average values of maximum dental arches opening were almost identical *P* = 0.495 (Figure 3(a)). On the other hand, in the group of patients with an articular-related ailment, a statistically significant difference was observed *P* = 0.009. As compared to healthy patients, it turns out that both in people suffering due to articular-related dysfunction as well as muscle, we are dealing with substantial reduction in the scope mandible abduction, and this reduction is particularly noticeable in patients with incorrectly functioning temporomandibular joints (Figure 3(b)). This limitation is the highest in patients with articular conditions and results directly from a considerable reduction in the length of articular route *S*_*S*_. Knowledge of the aforementioned parameters may be important when selecting the mode of therapeutic conduct, because in the case of confirmed muscle etiology of the dysfunction, a satisfactory therapeutic effect may be achieved using correctly performed massages [32].

The physiological scope of the condyloid process head movement in an arrow plane, recorded during mandible abduction, should be within 10 to 16 mm, which is said [33–35]. Similar results were obtained in the paper [36] where research was conducted on the group of 21 women aged 20 to 24. Additionally, this research was completed with the use of an electronic facebow Gnathohexograph JM-100. The obtained results indicate that the average value of dental arches opening 41.1 ± 3.5 mm corresponds to the average articular route of the condyloid process equal to ca, 12.8 ± 2.8 mm. At this point, it is worth mentioning the fact that the scopes of variability of recorded parameters were accordingly within 35.6 to 50.9 mm with regard to maximum dental arches opening and 8.1 to 19.2 mm in the case of articular route of condyloid processes. Studies on the length of articular route were also the object of publications [37]. In this study, a population of 25 people was studied. The research was conducted also with the application of an optoelectronic facebow Gnathohexograph JM-100 and the obtained results indicated that the average length of articular route is 14.16 mm. In cephalographic studies carried out by Muto and Kanazawa [38], the length of articular routes was recorded at the level of 20.5 mm for men and 18.1 mm for women. These values significantly exceeded the boundaries set out in the papers [33–35]. At this point, it is worth mentioning the fact that rigorous marking out boundaries between correct mastication organ movement and disturbed does not work in the clinical practice. To confirm the above it is worth mentioning the indications included in the paper [39], where attention was paid to the fact that exceeding the scopes deemed to be the standard is not always a sign of dysfunction. It is considered that this kind of disorders is symptoms of excessive ligaments slackening. Additionally, the disorders may be inborn or acquired. Acquired disorders undoubtedly prove ligaments pathology, while inborn should be regarded as a normal condition. Partial confirmation of this thesis can be obtained by analyzing the high variability of these parameters in a group of healthy people.

Statistical analyses carried out in this paper on the group of 13 healthy people indicate that the length of articular route covered by condyloid process heads assumes on average 39 ± 7% (mean ± SD) of maximum distance measured between the edges of central incisors. This ratio is much closer to the information provided in the paper by Ioi et al. [40] than the one suggested in the publications [33–35]. In the case of persons with mastication organ incorrect functioning, relation of articular route to the maximum opening gap of dental arches reached accordingly the level of 44 ± 11% in the case of disorders caused by improper muscles operation and 35 ± 11% in people with dysfunction caused by temporomandibular joints operation. At this point it is required to specify clearly and expressly that the gender of the patients did not have significant effect on the computed relations, since statistics values amounted to, accordingly, *P* = 0.467 in the case of muscle disorder and *P* = 0.479 in the case of articular dysfunction. In the light of the research conducted the relation of articular route length to the maximum dental arches opening proves to be an important indicator enabling to approximately determine the source of dysfunction.

On the basis of data distribution it may be concluded that there is close correlation between the length of the average articular route and the size of an opening gap. Additionally, most often listed length of condyloid process route ranges from 10 to 25 mm. This range corresponds to the opening gap measured between the edges of incisors contained within 30 to 50 mm. Values which are significantly different from average relate to individual people and are an insignificant percentage of the patients. Diagram (Figure 5(b)) compares statistical data characterising the relation of average length of articular route to the maximum size of an opening gap. The presented data show that the length of articular route is, on average, ca. 0.4 of the opening gap value expressed in millimetres. Variations of this coefficient in the group of healthy people were insignificant and contained in the range of 0.3 to 0.4. Numeric data which are significantly different from the mentioned were recorded in both groups of patients. Additionally, the largest discrepancies were observed in people with articular ailment, where extreme values ranged 0.06 to 0.69. Low values undoubtedly prove significant impairment of mobility in joint.

The presence of acoustic noises in temporomandibular joints is one of the symptoms of their incorrect operation [41, 42]. The external symptoms of these irregularities are various kinds of trajectory deviations recorded during mandible movements. In the case of ideally symmetric operation, the length of articular route of the left condyloid process *S*_*L*_ should be equal to the length of the right condyloid process route *S*_*P*_. With such an assumption as the starting point, a coefficient of adjusting measurement data for patients not reporting any disorders in the functioning of mastication organ were calculated (*R* ^2^ = 0.722). It should be borne in mind that comparable scopes of the length of articular routes *S*_*L*_ and *S*_*P*_ are not a sufficient condition to state proper operation of the mastication organ. The results of the conducted research indicate that correct mastication organ functioning exists when the length of both articular routes are comparable and in addition their values are within 13 to 23 mm. All the other cases can be treated as a sign of disorder. Additionally, the length of articular routes, whose value is within 0 to 13 mm, in the surveyed population where the result of incorrect operation of the joints. On the contrary, ones whose values were greater than 25 mm may prove disorder caused by improper muscular functioning (Figure 6(b)). It is confirmed by results of clinical tests also presented in Figure 5(a). The length of average articular route to be covered by condyloid process heads significantly determines the size of opening gap *Y* (Figure 7(a)). In the case of comparing measurement data, it is also possible to initially diagnose the reasons causing improper operation of the mastication organ. The studied parameter has an important diagnostic feature, since the analysis of location of item on a plane *S*_*L*_/*S*_*P*_ − *Y*, enables with a very high degree of probability to indicate the side responsible for the dysfunction.

The inclination angle of the articular route assessment* SCI* was also the subject matter of work [43], where a group of 4 men and 6 women was studied. From the studied population two groups were selected:* A—the group of the functional occlusal clutch* and* B—the group of the tray clutch.* The obtained results indicate that the average values of inclination angle of the articular route* SCI* are within the range of around 34.7 to 41.8 (group B) and from 35.6 to 42.8 (group A) and are similar to ones obtained by the authors of this study. The subject matter of the research, whose results were published in work [44] was comparison of the inclination angle of the articular route, measured by means of two various facebows. One of them was Gerber Dynamic Facebow, the second an electronic facebow Arcus Digma II. The research was conducted on a group of 35 women, aged 18 to 35. The average values of inclination angle of the articular route recorded with the ARCUSdigma II device for the right joint amounted to 33.1 ± 10.58 (mean ± SD) and left 32.4 ± 13.93. These angles are on average 13 larger as compared to the data recorded using the Gerber Dynamic Facebow (the right joint: 20.1 ± 9.94, the left joint: 19.4 ± 9.4). The obtained average values of inclination angle of the articular route* SCI*_*S*_ are consistent (Figure 8(a)) with the measurements conducted with the use of the electronic ARCUSdigma II facebow. In particular, studied groups they assume accordingly the values 27.8 ± 12.27 for the healthy group, 33.3 ± 12.47 in the group with troubles of articular origin, and 30.91 ± 10.87 for the group of patients with muscles functions disorders. It is worth bearing in mind the fact that the inclination angle of the articular route does not other words, along with ageing of the body its value is flattened, which indirectly may indicate geometrical shape of articular tubercle.

## 6. Conclusions

The ratio of the length of the articular route to opening gap of dental arches may be a significant indicator, characterising the degree of dysfunction intensification enabling initial diagnosis of the source of the human mastication organ incorrect functioning. An undoubtedly significant parameter informing the dentist about the location, that is, on which side there is the source of disorder is the ratio defined as the relation of the right length to the left of the articular route. In the case of proper mastication organ functioning, the ratio should be close to homogeneity. It seems that such research should be still continued in order to define clear criteria on the basis of which it will be possible to efficiently and effectively formulate the diagnosis. It seems that you should continue to pursue this kind of research, in order to define clear criteria on the basis of which it will be possible to formulate efficient and effective diagnosis. It should first of all determine the patterns characteristic of healthy people, which could become the reference criteria. Such actions were not subject of this research. The authors are aware that a group of 13 healthy subjects is too small to carry such an assumption.
